# Psychometric properties of Farsi version of the resilience scale (CD-RISC) and its role in predicting aggression among Iranian athletic adolescent girls

**DOI:** 10.1186/s40359-022-00852-2

**Published:** 2022-06-02

**Authors:** Roghieh Nooripour, Simin Hoseinian, Yaghoob Vakili, Nikzad Ghanbari, Joshua J. Matacotta, Nazir Mozaffari, Hossein Ilanloo, Carl Lavie

**Affiliations:** 1 Department of Counseling, Faculty of Education and Psychology, Alzahra University, Tehran, Iran; 2 Golestan Research Center of Psychiatry, Golestan University of Medical Sciences, Gorgan, Iran; 3 Faculty of Education and Psychology, Shahid Beheshti University (SBU), Tehran, Iran; 4 College of Health Sciences, Western University of Health Sciences, Pomona, USA; 5 Kharazmi University, Tehran, Iran; 6 John Ochsner Heart and Vascular Institute, Ochsner Clinical School-The University of Queensland School of Medicine, New Orleans, LA 70121 USA

**Keywords:** Resilience, CD-RISC, Aggression, Athletic girls, Adolescents

## Abstract

The sport presents an opportunity for young people to experience the joys of success and cope with setbacks to develop resilient behaviors. However, there is a lack of clarity about how sport can cultivate resilience, particularly among adolescent girls. This study investigated the psychometric properties of Farsi version of the Resilience Scale (CD-RISC) and its role in predicting aggression in Iranian athletic adolescent girls. The method of the present study was cross-sectional. The population of this study was Iranian athletic adolescent girls, and 475 Iranian athletic adolescent girls were selected through the convenience sampling method. The participants completed the Resilience Scale (CD-RISC), Quality of Mindfulness, General Self-efficacy (GSE), Alexithymia, and Aggression Scale. The CD-RISC’s psychometric properties were analyzed using confirmatory factor analysis, while reliability was tested using Cronbach’s alpha. Discriminant validity was measured by examining the relationship with alexithymia, and convergent validity was assessed with the quality of mindfulness and GSE. In addition, multiple regression analysis was conducted on the prediction of aggression by the CD-RISC subscales. The five-factor structure provided a good fit for the data. CD-RISC had significant negative associations with alexithymia, and there was a significant positive correlation between CD-RISC and Quality of Mindfulness, GSE. The results indicate that CD-RISC significantly predicts aggression in athletic adolescent girls. The CD-RISC has good validity for athletic adolescent girls in Iran and can be used in psychological evaluations in the Iranian context. CD-RISC significantly predicts aggression among athletic adolescent girls.

## Introduction

One of the athletes’ primary objectives is to develop their abilities to increase their commitment and sports performance. However, it is also necessary for them to consolidate the habits of a healthy lifestyle and fully develop their physical, cognitive, and social capacities [1, 2]. One of the crucial factors that affect sports experiences is resilience, which is controlled by various factors such as support, strategies, self-concept, motivation, and the athlete’s feelings [3]. In stressful competitions, resilience helps athletes increase their chances of success by enduring adversity and adapting positively to bitter experiences [4].

As one of the most important psychological factors, resilience helps adolescents adjust to adverse circumstances and increase adolescent well-being by using positive reinforcement strategies [5, 6]. Resilience can also show positive results, despite severe threats to adaptation, growth, or the ability to maintain, recover, or improve mental health after life’s challenges [7]. Resilience is considered a fundamental skill as it influences the development of athletic ability and personal level and is directly related to students’ mental health and psychological well-being [8]. Resilience protects young athletes in stressful and difficult competitions and prevents many problems [9]. In short, resilience contributes to the evolution of the exhaustive process of behavioral, social, and emotional adaptation [10].

Since resilient adolescents exhibit fewer harmful behaviors and have a higher mental health score, it makes sense to examine mental health concerning resilience so that adolescents can have better mental health by solving problems and adapting to the environment [11]. This point is crucial for athletes and coaches to have a favorable and stable mental state during competition. When a young athlete is exposed to adversity and stress, these resources help maintain balance [12]. According to research, resilience facilitates recovery from problems and increases the quality of coping styles of athletes in the face of future stressors [13].

Building resilience has been studied for decades but has only recently been explored in the sports field [14]. Athletes need to use and optimize various protective factors to deal with the stressful aspects of competition, sports environment, or daily life [15]. Resilient athletes had more ability to deal with problems, while non-resilient athletes used less active coping. Secades et al. [16] interviewed ten current or former academic and professional athletes and, through inductive analysis, identified five broad dimensions to describe the resilience experience: size and duration, confusion, socio-cultural influences, personal resources, and positive outcomes.

On the other hand, resilience increases a person’s ability to adapt and deal constructively with challenging situations. Conversely, aggression leads to intolerance and destructive ways of coping with complex problems based on this concept; the two variables are inversely related [17]. Resilience is a trait that helps regulate emotions, reinforce behaviors, promote healthy coping mechanisms, and maintain healthy interpersonal relationships. These later contribute to a lower likelihood and reduction of aggressive manifestations [18]. Therefore, looking at aggression is one of the most fruitful researches due to the emotional responses, which can affect the performance of athletes [19].

The Connor–Davidson Resilience Scale (CD-RISC), a 25-item questionnaire, assesses resilience. It has a five-point Likert scale that items could be used to calculate scores ranging from 0 to 100 when calculating the scores for these items. Connor and Davidson identified five dimensions of resilience: personal competence, high standards, and tenacity; trust in one’s instincts and tolerance of negative affect and strengthening effects of stress; positive acceptance of change and secure relationships; control; and spiritual influences [20].

However, when applied to a new context, there is a need to explore the validity of CD-RISC structure, differentiation, and prediction. For example, the five-factor structure found in the original study [20] was not repeated in subsequent investigations. While some studies showed two models [21], three [22], or four [23], most of the identified studies showed a one-dimensional structure of the scale, sometimes only 22 [24, 25] or ten items [26, 27]. This scale has already been used in a population of Spanish adolescents [28], Korean students, nurses and firefighters [29], French dental and medical students [30], Portuguese population [31], Indian students [32], people with diabetes in China [33] and even Iranian students [34]. It can be argued that the structure of CD-RISC is at least partially dependent on the context and population in which it is administered. There have been no validation studies of CD-RISC in Iranian athletic adolescent girls.

So there has been a variety of studies reporting the factor structures of the Connor–Davidson Resilience Scale (CD-RISC), which led to the present study’s objective to evaluate the psychometric properties of the Farsi version of the CD-RISC among Iranian athletic adolescent girls. The second objective was to test the relationships between CD-RISC and quality of mindfulness, general self-efficacy, and alexithymia to determine the convergent and discriminant validity. Then, we investigated whether CD-RISC predicts aggression among participants.

## Methods

This cross-sectional study was conducted in Iran between December 2018 and January 2019, selecting 475 Iranian athletic volunteers’ adolescent girls through a convenience sampling method. The STROBE checklist for cross-sectional studies was followed to improve methodological rigor [35], and for the general group, 125 general Iranian adolescent girls were selected using the convenience sampling method. So, the final research sample was 600 Iranian adolescent girls in the data analysis.

The data collection method included collecting the answers provided to the research questionnaires via the internet in Iran. Platforms included Instagram, Telegram, WhatsApp, Internet ads, and e-mails in Iran.

Participants in this study were required to meet the following criteria: (1) Students need to be in the high school age group (approximately 13–17-years-old) to meet the criteria related to Erik Erikson's theory of psychosocial development [36], (2) Participants must read and speak Farsi fluently and residence of Iran, (3) Students with no disabilities (e.g., motor, visual, hearing, intellectual or social impairments); and students not suffering from mental or cognitive disorders, (4) students who have not completed in surgery that prevent them from being physically active for the last six months, (5) a written and informed consent, (6) regardless of a particular sport and exercise regularly at the club at least two days a week, (7) Have at least one year of experience in training for their particular sport.

The following exclusion criteria were used in the current study: (1) athletes taking medication to treat injuries or illnesses, (2) the athletes who were in the process of losing or gaining weight at the same time, and finally, students with mental health issues such as anxiety, stress, or undiagnosed depression would need to have written permission from their teachers and parents before taking part in the survey otherwise, they would be considered as exclusion criteria for the study.

The questionnaires were self-administered so participants could check their responses before answering. There was no need to write a name, and that participation in the research was according to their personal preference due to the use of an online survey and setting the necessary answers for each item.

### Measures

#### The Connor–Davidson resilience scale (CD-RISC)

This is a self-report scale developed by Connor and Davidson in 2003. The scale is a 25-item instrument that measures resilience structure in a five-point Likert-type from zero to four, zero being the minimum resilience score [20]. Therefore, the range of test scores is between 0 and 100. Higher scores indicate higher resilience of the subject. The factor analysis results show that this test has five factors: Personal competence, high standards, and tenacity; Trust in one’s instincts, tolerance of negative affect and strengthening effects of stress; Positive acceptance of change and secure relationships; Control and Spiritual influences.CD-RISC authors found that test-retest reliability (ICC = 0.87) and internal consistency (α = 0.89) were acceptable [20]. Additionally, Yu et al. [22] reported a strong internal consistency coefficient (α = 0.89) for their sample of Chinese adolescents.

#### Quality of mindfulness

The Cognitive and Affective Mindfulness Scale-Revised (CAMS-R) [37] is a 12-item scale measuring everyday mindfulness. It focuses on how individuals are aware of their thoughts and feelings and does not require meditation training. Items are rated on a 4-point Likert scale from one (not at all) to four (almost always). Mohsenabadi et al. [38] also reported a Cronbach’s alpha of 0.80 in Iran.

#### General self-efficacy scale (GSE)

The scale was developed by Schwarzer and Jerusalem in 1979 and revised in 1981 into ten items, all general measuring self-efficacy. The assessment is based on a four-point Likert scale ranging from one to four. The Cronbach’s alpha coefficient of this scale was 0.82 [39]. In Iran, the Cronbach’s alpha coefficient of this scale was 0.81 [40].

#### Alexithymia

The Toronto Alexithymia Scale-2 [41] is a 20-item self-report measure of alexithymia. Items are rated on a Likert scale ranging from one (strongly disagree) to five (strongly agree). The scale’s internal consistency by Cronbach’s alpha for the total score was 0.72 [42].

#### The aggression scale

It is a measure with 11 indicators to assess aggressive behaviors -the responses in each indicator range from 0 (times) to 6 (or more often) [43]. The Farsi version of the aggression scale was validated in Iran and showed good reliability with a Cronbach’s alpha of 0.87 [44].

### Procedure

Before the survey, informed consent was obtained from participants and their parents/legal guardians. This study strictly protected participants’ confidentiality. Therefore obtaining informed consent was a priority. Consenting participants (and their parents/legal guardians) and questionnaires were then entered into *Google Forms,* and the link was sent to social networks to be completed online by the respondents. The survey collected demographic characteristics, including age and sport type. The survey also included an information sheet reminding participants of their voluntary and anonymous participation, and it is coded to do the test-retest reliability step. The respondents had the right to choose whether to join the study and provide information or withdraw—inter-rater agreement measured by Cohen’s kappa coefficient (K = 0.76) indicates acceptable agreement.

The data collection period began in December 2018 and January 2019. The online questionnaires took an average of 25 min to complete for each respondent. Also, the study procedure was approved by an ethical committee of Alzahra University (IR/12/12/1400). All procedures were carried out under applicable guidelines and regulations. Alongside the translated CD-RISC, the other scales used in this study were already translated into Farsi.

This research was divided into two phases: the instrument’s translation and the cultural adaptation technique, then analysis of its psychometric properties and the verification of its validity. The CD-RISC was translated into Farsi in the first phase using the back-translation technique. In this technique, one translation team translates the scale into Farsi, and then the second team translates it back into the original language. The translation accuracy was judged by closely matching the second team’s original version. However, as Hambleton [45] pointed out, this commonly used technique has shortcomings. They suggested that translators be proficient in both languages and familiar with both cultures. The quality of the translation has been assessed as to how it fits the initial text. Accordingly, two translators were contacted to help with the study. They worked independently, and no significant differences were found in the translation and expression of the items. The authors subsequently reached a consensus with the translators on both versions. Finally, some items were revised by a professor of English and other psychologists to make them more understandable and comprehensible to the target audience. Care was taken to ensure that the length of the items corresponded to the original scale. The authors then achieved agreements with the translators for the final version.

### Statistical analysis

The IBM SPSS Statistics 22.0 (IBM SPSS Statistics, Inc., Armonk, USA) was used to analyze demographic frequencies and measure the Pearson correlation between the CD-RISC with the quality of mindfulness and general self-efficacy and alexithymia. In addition, the confirmatory model Factor Analysis (CFA) was used. The internal consistency of the CD-RISC was determined using Cronbach’s alpha. In addition, a multiple regression analysis was used to predict aggression by the subscales of the CD-RISC.

### Preliminary analysis

Preliminary tests, such as data loss analysis, discarded data, and normality of the data were performed before the CFA. Discarded data were assessed by the Mahalanobis distance square with a significance level of 0.001 in AMOS software, and no discarded pieces of data were identified. Skewness (0.154–0.181) and Kurtosis (0.35–2.15) were used to test the normality assumption of the items. The normality of data is met when values range between ± 2 for skewness and ± 3 for Kurtosis [46], and results showed normal distribution of the data.

## Results

A total of 475 Iranian athletic adolescent girls participated in the survey. The participants' mean (SD) age was 15.68 (1.89). Regarding sports status, 246 (52.9%) of the participants were Individual sports, and 229 (47.1%) played team sports (see Table 1).

**Table 1:** Socio-demographic characteristic (*n* = 475)

	N	%	M	SD	t(df)	*p*
*Sport type*					1.99(473)	0.046
Individual sport	246	52.9	68.00	16.17		
Team sport	229	47.1	65.17	14.61		
*Age group*					0.911(3.471)	0.43
14	113	23.5	65.94	15.42		
15	166	35.8	68.20	15.56		
16	148	30.8	65.95	12.93		
17	48	9.9	65.02	21.57		

### Factor structure

The Kaiser-Meyer-Olkin (KMO) index was 0.908, above the recommended value of 0.6, and Bartlett’s test for sphericity reached statistical significance (× 2 = 2855.39, *p* ≤ 0.001) that the data were suitable for factor analysis. We conducted CFA on a sample of 475 athletic adolescent girls. The initial CFA began with five factors and 25 items. The final CFA showed five factors, 25-item solutions: the factor loadings and fit statistics (Table 2).

**Table 2:** CFA of Connor–Davidson resilience scale (CD-RISC)

Items	Factor loading	Eigen value			
		Total	% of variance	Cumulative %	Overall alpha
ITEM 1	0.43	9.377	37.509	37.509	0.912
ITEM 2	0.41	1.635	6.538	44.047	
ITEM 3	0.83	1.279	5.118	49.165	
ITEM 4	0.63	1.167	4.148	53.313	
ITEM 5	0.62	1.016	3.988	57.301	
ITEM 6	0.58	0.882	3.526	60.828	
ITEM 7	0.57	0.865	3.461	64.288	
ITEM 8	0.46	0.788	3.153	67.441	
ITEM 9	0.65	0.768	3.070	70.511	
ITEM 10	0.76	0.696	2.783	73.295	
ITEM 11	0.70	0.651	2.602	75.897	
ITEM 12	0.70	0.609	2.437	78.334	
ITEM 13	0.51	0.584	2.334	80.669	
ITEM 14	0.75	0.520	2.080	82.748	
ITEM 15	0.76	0.514	2.057	84.805	
ITEM 16	0.69	0.504	2.014	86.820	
ITEM 17	0.65	0.480	1.919	88.738	
ITEM 18	0.47	0.430	1.721	90.460	
ITEM 19	0.78	0.412	1.648	92.108	
ITEM 20	0.72	0.378	1.511	93.619	
ITEM 21	0.76	0.358	1.433	95.052	
ITEM 22	0.67	0.345	1.380	96.432	
ITEM 23	0.39	0.320	1.279	97.711	
ITEM 24	0.71	0.295	1.182	98.892	
ITEM 25	0.59	0.277	1.108	100.000	

**Fig. 1:**
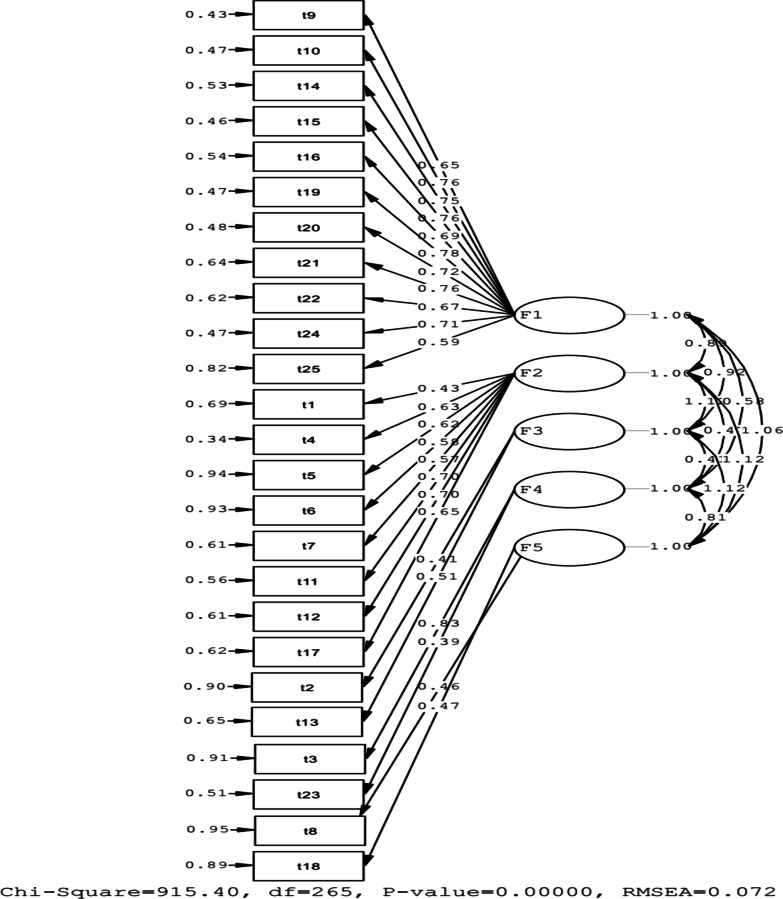
Measurement model of Connor–Davidson Resilience Scale (CD-RISC) among Iranian athletic adolescent girls. χ^2^(265) = 915.40, *p* > 0.001, χ^2^/df = 3.454, RMSEA (90% C.I.) = 0.072 (0.067–0.077). F1 = Personal Competence, High Standards, And Tenacity; F2 = Trust in One's Instincts and Tolerance of Negative Affect and Strengthening Effects of Stress; F3 = Positive Acceptance of Change and Secure Relationships; F4 = Control; F5 = Spiritual influences

The standardized factor loading for the five CD-RISC items can be seen in Fig. 1, where all the factor loadings were significant. Except for item 23, results demonstrated that the factor loading of item 23 was 0.39 (under 0.40), so this item could be omitted(and this is unnecessary, so it needs further investigation).

The CFA results for a five-factor structure are shown in Table 3. Model fit was estimated using the following fit indices: Root Mean Square Error of Approximation (RMSEA; criterion 0.08) and its confidence level of 90%, Standardized Root Mean Square Residual (SRMR; criterion 0.09), Comparative Fit Index (CFI; criterion 0.90), Normed Fit Index (NFI; criterion 0.90), Incremental Fit Index (IFI; criterion 0.90), and Relative Fit Index (RFI; criterion 0.90). The CFA results also showed that the five-factor structure fit data well. In the present study, fit indices of model were RMSEA = 0.072; SRMR = 0.051, RMR = 0.057, CFI = 0.97, NFI = 0.96, IFI = 0.97, RFI = 0.95. All items of loadings showed significant factor (Table 3).

**Table 3:** Confirmatory factor analysis (CFA) and fit indexes

model	RMSEA (CI 90%)	_sb_X^2^	RMR	SRMR	CFI	NFI	IFI	RFI
25 items	0.078 (0.067–0.077)	915.40	0.057	0.051	0.97	0.96	0.97	0.95

*RMSEA* Root mean square error of approximation; *RMR* Root mean square residual; *SRMR* Standardized RMR; *CFI* Comparative fit index; *NFI* Normed fit index; *IFI* Incremental fit index; *RFI* Relative fit index

### Face validity

This study evaluated its appropriateness, representativeness, readability, and clarity using face validity. Before collecting data from a large sample, cognitive interviews are helpful for researchers to clarify items, ensure adequate coverage of the content, and modify the questionnaire if any questions are unclear [47]. Twelve athletic adolescent girls participated in cognitive interviews to determine item complexity, vagueness, and comprehensibility of interview items. Twenty-five questions were ultimately compiled as a final scale. As a result, no changes had to be made to the Persian scale when designing the CD-RISC's final Persian version, as there were no unclear Persian terms.

### Internal consistency reliability

The internal consistency reliability of the Iranian Connor–Davidson Resilience Scale (CD-RISC) was assessed using Cronbach’s alpha for all participants and was 0.908. The internal consistency of the Iranian Connor–Davidson Resilience Scale (CD-RISC) was similar.

### Follow-up study and test-retest reliability

Temporal stability using a test-retest strategy in a small subsample of 105 participants in the main study was randomly selected and asked to complete the CD-RISC again after two weeks. The results showed that after this period, the calculated test-retest coefficient was 0.81(CI = 0.79–0.83).

### Convergent validity

The convergent validity of the CD-RISC was assessed by correlation with the Quality of Mindfulness and General Self-efficacy (GSE). Significant positive correlations of the CD-RISC subscales with Quality of Mindfulness and General Self-efficacy (GSE) ranging from 0.19 to 0.48 indicated acceptable convergent validity (Table 4).

**Table 4:** Pearson correlation between CD-RISC with quality of mindfulness, general self-efficacy (GSE), and alexithymia among participants

	F1	F2	F3	F4	F5	CD-RISC Total
Quality of Mindfulness	0.28^**^	0.23^**^	0.21^**^	0.19^**^	0.20^**^	0.31^**^
GSE	0.41^**^	0.37^**^	0.28^**^	0.23^**^	0.25^**^	0.44^**^
Alexithymia	− 0.31^**^	− 0.28^**^	− 0.22^**^	− 0.20^**^	− 0.19^**^	− 0.34^**^

^*^p < 0.05, ^**^p < 0.01

In Table 4, there were significant negative correlations between the subscales of CD-RISC and the alexithymia (ranging from − 0.19 to − 0.34) *p* < 0.01, indicating acceptable discriminant validity.

### Discriminant validity

As shown in Table 5, discriminant validity was calculated by comparing resiliency in general and athletic Iranian adolescent girls.

**Table 5:** Independent t test for comparing resiliency in athlete and general groups

Variable	Group	*N*	M	SD	t	df	*p*
Resiliency	Athlete	475	66.64	15.49	2.25	598	0.025
	General	125	63.21	1.77			

Results of the Independent t-test showed that there was significant difference [t(598) = 2.25, *p* ≤ 0.05] between groups, mean of resiliency in the athlete group (66.64) significantly higher than normal group (63.21). The significant difference between athletes and general Iranian girl adolescents was *p* < 0.05, indicating acceptable discriminant validity.

### Role of resilience in predicting aggression

Multiple regression analysis was used to predict aggression by subscales of CD-RISC among Iranian athlete girls (Table 6).

**Table 6:** Multiple regression analysis for prediction of aggression by subscales of CD-RISC. (*N* = 475)

	B	S. E	Beta	T	P	R	R^2^	F	P
Constant	42.352	3.123		6.347	0.001	0.457	0.208	19.64	0.001
F1	0.171	0.058	0.146	3.914	0.001				
F2	0.159	0.062	0.127	3.531	0.001				
F3	0.154	0.054	0.114	2.531	0.023				
F4	0.161	0.059	0.101	2.344	0.039				
F5	0.112	0.064	0.093	1.762	0.087				

Multiple linear regression was calculated to predict aggression based on the components of CD-RISC; the results showed that components of CD-RISC significantly predicted aggression in Iranian athletic adolescent girls (F (5, 470) = 19.64, *p* ≤ 0.001), with R^2^ of 0.21 (Table 6).

## Discussion

This study examined the Farsi version of the Connor–Davidson Resilience Scale (CD-RISC) and its role in predicting aggression in Iranian athletic adolescent girls. Findings showed no items had been removed in the current research, and all items have a high factor. The results are broadly consistent with previous studies that showed high internal consistency for the CD-RISC in clinical and general populations [22, 48–50] and in contrast to the findings of Giurcă [51], Velickovic [52] that some questions and factors were omitted. Our research showed that resilience scores in athletic adolescent girls were higher than in general girl adolescents. Codonhato et al. [53] showed that athletes are more resilient and better prepared to deal with challenges and stresses in the sports environment.

Consistent with Arpaci and Gundogan [54], Musil et al. [55], quality of mindfulness and CD-RISC had a positive correlation. In previous studies, such as Orkaizagirre‐Gómara et al. [56], and Tagay et al. [57], based on the results of this study, it is a positive correlation between CD-RISC and general self-efficacy. It is expected that an increase in resilience level will lead to an increase in general self-efficacy levels as adolescents with self-efficacy levels are more successful in difficult situations [57]. There is also a negative correlation between CD-RISC and alexithymia that is consistent with results in Chinese soldiers [58] and Iranian students [59]. Alexithymia is observed in people suffering from psychopathological disorders that lower patients' resilience [58, 60, 61]. Mikolajczak and Luminet [62] established that resilience has been related negatively with resilience. Resilience requires self-regulation of emotions and social support, both of which are lacking in individuals with a high level of alexithymia. Alexithymic individuals are unable to distinguish between emotional and physical sensations and show difficulties recognizing emotional distress [61].

Our research findings showed that resilience could predict aggression. Accordingly, athletic adolescent girls with higher resilience scores had lower aggression scores. Therefore, it is possible to focus on the resilience level of athletic adolescent girls and provide more appropriate psychological services for aggression control through proper facilities [63]. Adolescents are vulnerable to aggression, depression, and other disorders due to the blows they suffer early in life [64]. Increasing resilience is a valuable coping mechanism that helps people deal with stressful situations, and it allows people to make sense of hope and adapt to the environment and humans. Resilience helps athletic adolescent girls to withstand challenges and not be aggressive by regulating their emotions. In addition, resilient athletic adolescent girls and considerable control over their feelings reject aggression and avoid destructive and violent activities [64].

The present research tried to open discussion on the resilience behaviors that may help to raise awareness of sporting scenarios in athletic adolescent girls. There is no doubt that further research on this concept in different clinical and research settings is necessary. Since there was a relationship between resilience and aggression in this study, it is recommended that specific mechanisms in the sport that contribute to resilience should be further identified and investigated. Identifying these mechanisms will allow researchers to pursue other non-sport activities with some precise tools. Planning to increase resilience in athletic adolescent girls can play an essential role in reducing aggression and help authorities to prevent social harm and intervene promptly. Future research could examine the influence of family on resilience in adolescent athletes. We hope our decision provides enhanced consistency with existing work and permits researchers to continue evaluating the multidimensional nature of resilience.

## Limitations and recommendation

The present research findings should be interpreted in light of the study’s limitations. The study’s main limitation was that it was only conducted on athletic adolescent girls, which constituted the main limitation. At the same time, the various cultures and ethnicities present in the country should also be considered in further research. Another limitation, which relates to this point, is that the study is cross-sectional, suggesting that caution should be exercised when generalizing the conclusions. Moreover, it is recommended that the results of this study are used to develop educational packages that will promote the resilience of girls and women. Another limitation is the design of the study. While online data collection is convenient, it could be biased toward those with Internet access. As mentioned above, there is a need to develop a sport-specific measure of resilience. For example, developing a sport-specific measure of resilience that fully and accurately represents the process of resilience in sport is vital to accurately and reliably measure the function of resilience. This validation study is based on Classic Theory Test (CTT), and we suggest researchers perform Item Response Theory (IRT) in future research, which can provide some additional important information, i.e., item difficulty, discriminative ability, etc. What’s more, it will be helpful to address the factor-related issues in this study.

There should be a more systematic understanding of resilience as a state-trait mixed psychological variable that is amenable to intervention and can be used as a primary outcome in intervention trials. The subscale of positive acceptance of change measures state resilience by deciding whether the individual is competent and accepts themselves and life. These qualities demonstrate an individual’s capacity to cope and adapt to adversity. So resilience is now understood to be more of a dynamic process than a rigid individual characteristic [65]. Future longitudinal studies are necessary to determine whether this measure has long-term psychometric properties and predictive validity. It is also suggested that a comparative study be conducted in the clinical population (e.g., individuals with varying levels of depression, anxiety, etc.) and the boys’ population using this scale.

As there is no golden standard for defining and measuring resilience in athletic adolescent girls, field-specific and established instruments had to be used as anchors. So, this study provides some initial psychometric evidence that the CD-RISC-25 can be used to measure individual qualities in sports performers. Future researchers should consider possible antecedents of resilience in sports and measure items that consider all three factors (individual, social, and environmental). This information will enable us to predict which athletes will overcome adversity. Researchers and practitioners can both benefit from this type of information.

Despite these limitations, our results are helpful in that they show that CD-RISC could be used as a complementary instrument to assess resilience in Iranian athletic adolescent girls. We believe that the Farsi version of CD-RISC will provide researchers with a reliable and valid method for measuring multiple aspects of resilience that will facilitate the inclusion of this measure in study protocols and reduce the burden on participants.

## Conclusions

In conclusion, the 25-item CD-RISC showed good psychometric properties in assessing resiliency, and also, CD-RISC significantly predicts aggression among athletic adolescent girls. Moreover, this instrument and other psychological assessment instruments provide a suitable platform for psychologists, clinicians, and researchers involved in adolescent and sport-related activities in clinical and research settings.

## Data Availability

The datasets generated during and analyzed during the current study are available from the corresponding author on reasonable request.
